# Supplementary material for: Deep learning-based spatial immunoprofiling of multiplex immunofluorescence images distinguishes tuberculosis disease states in diversity outbred mice
Source: PLoS One. 2026 Sep 25;21(9):e0358334. doi: 10.1371/journal.pone.0358334 (PMC13614646; doi:10.1371/journal.pone.0358334)
Supplement: Supplementary file 1 [file pone.0358334.s001.docx]

| A. CD4 | B. CD8a |
| --- | --- |
|  |  |
| C. CD19 | D. IBA1 |
|  |  |
| E. EPCAM |  |
|  |  |

**Supplementary Figure S1. Density of immune cell populations per unit granuloma area across TB disease states (10% boundary shrinkage applied).** Box plots show density of cells, reported as density per unit granuloma area for **(A)** CD4+ T cells, **(B)** CD8a+ T cells, **(C)** CD19+ B cells, **(D)** IBA1+ macrophages, and **(E)** EpCAM+ epithelial cells across three disease groups: Progressor, Asymptomatic, and Controller. Each dot represents one granuloma; box plots show median, interquartile range, and whiskers extending to 1.5× IQR.

| A. CD4 | B. CD8a |
| --- | --- |
|  |  |
| C. CD19 | D. IBA1 |
|  |  |
| E. EPCAM |  |
|  |  |

**Supplementary Figure S2. Total numbers of immune cells in granulomas across TB disease states (10% boundary shrinkage applied).** Box plots showing cell abundance for **(A)** CD4+ T cells, **(B)** CD8a+ T cells, **(C)** CD19+ B cells, **(D)** IBA1+ macrophages, and **(E)** EpCAM+ epithelial cells across three disease groups: Progressor, Asymptomatic TB, and Controller. Each dot (A-E) represents one granuloma; box plots show median, interquartile range, and whiskers extending to 1.5× IQR.
