# Supplementary material for: Deep learning-based spatial immunoprofiling of multiplex immunofluorescence images distinguishes tuberculosis disease states in diversity outbred mice
Source: PLoS One. 2026 Sep 25;21(9):e0358334. doi: 10.1371/journal.pone.0358334 (PMC13614646; doi:10.1371/journal.pone.0358334)
Supplement: Supplementary file 2 [file pone.0358334.s003.docx]

Dear Editor,

We appreciate the thoughtful critique of the mouse model of tuberculosis and acknowledge the limitation, particularly regarding inbred mice, like the C57BL/6 strain, which models one genotype’s response to *M. tuberculosis* infection and does not recapitulate human TB genotypic or phenotypic heterogeneity. However, the Diversity Outbred (DO) mouse population, which we use here, has similar genetic diversity as the human population within and between individuals. This genetic architecture, and phenotypic responses to infections, are much more heterogeneous than individual inbred strains. To establish the scientific basis for our work, we refer to two peer-reviewed publications: Ahmed et al. (Science Translational Medicine, 2020; 12(528):eaay0233) demonstrated that immune and inflammatory pathways of TB disease states are conserved across nonclinical animal models (DO mice, non-human primates, and humans), in particular a 16-gene human TB signature that was highly expressed in DO progressor mice. Koyuncu et al. (Infection and Immunity, 2024; 92(7):e00263-23) provided a comprehensive comparison of infection states, clinical aspects, and granuloma architecture in humans versus DO mice, summarized in Table 1, demonstrating that DO mice share immunological and granuloma features of human TB.

We audited our manuscript for claims inappropriately equating DO mouse characteristics with human TB, specifically searching for human TB diagnostic terms and disease states that have been described in the research literature: “primary TB”, “fulminant TB”, “post-primary TB”, “latent infection,” “latent TB”, “resistors,” "incipient," and "subclinical" to double check whether these terms were interchangeably to refer to any of the DO mouse disease states. We found no such claims of equivalence; these terms appear only in our introduction (describing human TB) and citations, not characterizing mouse outcomes. We apologize if any presentation created confusion regarding biological equivalence. However, as established by Ahmed et al. (2020) and Koyuncu et al. (2024), documented similarities exist in immune and inflammatory responses, granuloma features, microbiological cultivability, and clinical observations of biological determinants of disease outcome between DO mice and humans. We reiterate that peer-reviewed publications document conserved similarities between species, and acknowledge that not all *M. tuberculosis* infection states in humans have been identified in the Diversity Outbred mouse population. The information gained from using such animal models may still provide useful knowledge and insight into pathways that contribute to control of *M. tuberculosis* infection and pathogenesis of TB disease.

Detailed responses to the Reviewers are below.

Sincerely,
Usama Sajjad, on behalf of the authors

**Reviewer #1:** In this review, the authors have devised artificial intelligence and machine learning techniques to analyze Tuberculous granulomas for better understanding of disease progression and prediction. The study highlights the significant bottlenecks in understanding Tuberculosis. However, I have following concerns regarding the manuscript,
1. **The authors have not included any functional markers (e.g., M1/M2 polarization, TNFα, IL1β) for spatial localization and their significance? This is very important for coming to any conclusion.**

We thank the reviewer for this important and valid observation. We acknowledge that the absence of functional markers such as M1/M2 polarization indicators, TNFα, and IL-1β for spatial localization is a limitation of the current study, and we have added this to the limitations section of the Discussion accordingly. The primary aim of this study was to develop and validate an automated computational framework for granuloma segmentation, disease-state classification, and spatial quantification of immune cell populations from mIF images, rather than to provide a comprehensive functional characterization of immune responses across disease states. The functional immune and inflammatory responses, bacterial control capacities, and molecular signatures of the Progressor, Asymptomatic, and Controller phenotypes have been extensively studied in our prior work and by others, using protein detection methods, gene expression profiling, and pathway analysis. Specifically, lung cytokines and chemokines including TNFα, IFN-γ, IL-10, CXCL1, CXCL2, CXCL5, MMP8, S100A8, and VEGF have been quantified and analyzed across disease states in our previously published studies [1-6]. The immune and inflammatory correlates of these disease phenotypes have also been validated by independent groups [7, 8].

The current study builds on this established functional foundation by adding a spatial dimension, quantifying where immune cells are located within granulomas and how those spatial relationships differ across disease states. The inclusion of functional markers for spatial localization in future mIF panels is an important direction that we fully agree with and acknowledge as a priority for subsequent work.

**2. Given the subjectivity in granuloma boundary definition, how do the authors ensure annotation consistency across samples?**

We thank the reviewer for this insightful question. The authors ensured annotation consistency by grounding the entire segmentation pipeline in expert pathological knowledge. Granuloma boundary annotations were delineated by a board-certified pathologist, providing biologically accurate and consistent ground truth segmentation masks for model training. Importantly, deep learning segmentation models learn by identifying recurring patterns in training data, meaning that inconsistent or contradictory annotations across samples would prevent the model from learning a coherent decision boundary, ultimately resulting in poor performance on unseen data. The IF-UNet++ model was then trained on these expert-defined granuloma annotations and quantitatively evaluated, achieving an IoU of 90.57% on the test set, demonstrating that the model successfully learned to replicate pathologist-level accuracy across diverse disease states and tissue morphologies. The fact that IF-UNet++ achieves high IoU on samples never seen during training is therefore not only a measure of model accuracy but also indirect quantitative evidence that the underlying annotations were consistent and reproducible across the dataset. Beyond quantitative evaluation, the segmentation results were mapped back to the original mIF images and qualitatively reviewed by a board-certified pathologist, who confirmed that the predicted granuloma boundaries were spatially concordant with true granulomatous structures across all disease phenotypes. Taken together, the expert-guided annotation, strong quantitative performance, and qualitative pathologist validation form a coherent and mutually reinforcing validation strategy that collectively addresses the inherent subjectivity in granuloma boundary definition. We have expanded this into section 3.1.
**3. Did the authors quantitatively compare predicted vs. true granuloma morphology (e.g., size, shape descriptors.**

We performed the quantitative comparison of predicted versus true granuloma morphology using the Intersection over Union (IoU) metric, which serves as a comprehensive surrogate for all aspects of granuloma morphological accuracy, including size, shape, spatial extent, and boundary fidelity.

IoU is computed by dividing the area of overlap between the predicted segmentation mask and the ground truth mask by the area of their union. If the predicted boundary is too large or too small, the size mismatch directly penalizes the score. If the shape is incorrect, the misaligned boundaries reduce the shared region and lower the IoU. If the spatial location is off, the same penalty applies. In this way, IoU implicitly encodes agreement in size, shape, location, and boundary fidelity all within a single unified metric, making it a comprehensive surrogate for granuloma morphological accuracy without requiring each descriptor to be reported separately.

Our IF-UNet++ model achieved an IoU of 93.22% on the training set and 90.57% on the test set, demonstrating strong spatial and morphological agreement with the pathologist-defined ground truth across diverse disease states and tissue morphologies. We therefore believe that the high IoU scores reported in our study provide sufficient evidence of accurate granuloma geometry capture, and that these results are adequate for supporting the reliability of our downstream spatial and disease-state analyses. We have further clarified it in section 3.1.

**4. How does segmentation quality impact downstream biological interpretation or feature extraction.**

We thank the reviewer for highlighting the importance of segmentation quality with respect to downstream biological interpretation and feature extraction. To directly address this question, we introduced controlled perturbation by deliberately shrinking the granuloma boundary by 10% of its area and recomputed downstream cellular metrics, including immune cell density per unit granuloma area and total immune cell counts across all cell types (Supplementary Figure S1, S2).

Our findings demonstrate that although the absolute value of cell counts and densities change because of the boundary perturbation, the overall biological trends across the three disease groups, namely Progressor, Asymptomatic, and Controller, remain consistent with our original results. This indicates that our downstream biological interpretations are robust to minor segmentation inaccuracies and are not artifacts of precise boundary placement. The biological signatures we report, such as higher CD4+ and CD8a+ T cell densities in Asymptomatic mice and elevated total cell counts in Controllers, are preserved under this perturbation, supporting the stability and reliability of our conclusions.

We have added the corresponding plots as Supplementary Figures S1 and S2, showing density of immune cell populations per unit granuloma area and total immune cell numbers respectively, both computed under the 10% boundary shrinkage condition, for all five cell types across the three disease states. These supplementary results collectively demonstrate that our framework produces conclusions that are resilient to small variations in segmentation quality.

| A. CD4 | B. CD8a |
| --- | --- |
|  |  |
| C. CD19 | D. IBA1 |
|  |  |
| E. EPCAM |  |
|  |  |

**Supplementary Figure S1. Density of immune cell populations per unit granuloma area across TB disease states (10% boundary shrinkage applied).** Box plots show density of cells, reported as density per unit granuloma area for **(A)** CD4+ T cells, **(B)** CD8a+ T cells, **(C)** CD19+ B cells, **(D)** IBA1+ macrophages, and **(E)** EpCAM+ epithelial cells across three disease groups: Progressor, Asymptomatic, and Controller. Each dot represents one granuloma; box plots show median, interquartile range, and whiskers extending to 1.5× IQR.

| A. CD4 | B. CD8a |
| --- | --- |
|  |  |
| C. CD19 | D. IBA1 |
|  |  |
| E. EPCAM |  |
|  |  |

**Supplementary Figure S2. Total numbers of immune cells in granulomas across TB disease states (10% boundary shrinkage applied).** Box plots showing cell abundance for **(A)** CD4+ T cells, **(B)** CD8a+ T cells, **(C)** CD19+ B cells, **(D)** IBA1+ macrophages, and **(E)** EpCAM+ epithelial cells across three disease groups: Progressor, Asymptomatic TB, and Controller. Each dot (4A-4E) represents one granuloma; box plots show median, interquartile range, and whiskers extending to 1.5× IQR.
**5. The classification labels are defined by survival: Progressor (<60 days) vs. Controller (>60 days). Is this threshold biologically justified or empirically chosen? Also, how stable are these labels across cohorts? Could survival-based labeling introduce bias unrelated to granuloma morphology?**

The 60-day survival threshold is biologically justified and empirically well-supported across our cohorts. As described in our previously published work [5], across nearly 1,000 total DO mice spanning cohorts of approximately 125 mice each, 25–30% consistently develop pulmonary TB morbidity requiring euthanasia prior to day 60, with a peak morbidity wave occurring between days 25–35 post-infection. The remaining 70–75% are asymptomatic through this 60-day window and many remain so substantially beyond that, up to nearly 2 years. This survival pattern across independent cohorts demonstrates that the 60-day threshold captures a real and stable biological boundary between acute disease susceptibility and bacterial control, rather than an arbitrary cutoff.

We acknowledge that survival-based labeling could theoretically introduce bias if mice developed morbidity due to non-TB disease. This was minimized by examining all mice through multiple approaches to confirm that morbidity was related to pulmonary TB and not to other co-morbidities. Mice with clear co-morbidities were excluded and are not included in any analyses presented here. The detailed methods used to ensure that morbidity was attributable to TB rather than other disease are described in the Quantification of TB-related Traits (Phenotyping) section of our previously published work [5].

**6. Please clarify how distances were computed from pixel-to-micron.**

We appreciate the reviewer raising this point. Our WSIs have a spatial resolution of 0.5 microns per pixel (mpp), meaning each pixel corresponds to a physical length of 0.5 µm in tissue space. To ensure biologically meaningful spatial analysis, all pixel-based distance measurements were converted to physical units using this linear scaling factor. Specifically, to identify cell populations within a 75 µm radius, we converted the radius from microns to pixels using the formula: RadiusInPixels = RadiusInMicrons / mpp = 75 / 0.5 = 150 pixels. This conversion was applied uniformly across all WSIs to maintain consistency in defining spatial neighborhoods. The resulting 150-pixel radius was then used to query and count cell populations within that spatial boundary for each cell of interest. This approach ensures that our proximity-based analysis reflects true physical distances in tissue, independent of any pixel-resolution variability across imaging sessions. We have clarified it in the manuscript section 3.5.
**7. How were bronchiolar epithelial cells defined? Based on EpCAM positivity alone? Were airway structures (bronchioles) explicitly segmented or annotated? EpCAM+ cells may include non-bronchiolar epithelial cells unless spatially constrained.**

EpCAM positive cells were confirmed to be bronchiolar epithelial cells based on the anatomic structure highlighted by board certified pathologists. We have clarified it in section 3.2.

**8. Why would closer B-cell proximity to bronchiolar epithelium associated with asymptomatic infection?**

This is a good question that we did not address directly here but could be an association or a mechanism. A plausible hypothesis is that B-cells in that region are differentiating into plasma cells that may secrete IgA into the airways or IgG into the blood stream that may help the host control infection in the lungs or limit dissemination.

**9. Were analyses stratified by: Granuloma size? Necrotic vs. non-necrotic lesions? Spatial organization can vary dramatically with granuloma stage.**

Analyses were stratified by host disease state. We agree that stratification by other means is a good idea and spatial organization can vary by granuloma stage. These types of questions may be better answered by separate experiments using inbred strains of mice at pre-defined time points.

**Reviewer #2:** Authors investigated the spatial distribution of lung granulomas in different clinical stages of tuberculosis disease using a mouse model. The authors developed a novel automated model for granuloma segmentation in lung tissues. The study demonstrates distinct spatial distributions of CD4+ and CD8a+ positive cells during the asymptomatic stage of disease. In addition, the LLM model decoded complex cellular patterns within granulomas, including balanced immune responses in asymptomatic mice, dysfunctional responses with low cellularity in acute TB, and increased immune cell accumulation during chronic disease. Overall, the findings are interesting and contribute valuable insights into the immunopathology of tuberculosis.
**Major Comments:
1. The authors performed multiplex immunofluorescence imaging of lung sections. Did the authors use sequential staining on the same tissue sections after stripping the previous antibodies/signals? This is not clearly described in the Methods section.**

We use tyramide signaling amplification for our multiplex immunofluorescence images. In each staining cycle, a primary-secondary antibody complex was applied, followed by covalent deposition of the fluorophore onto the tissue via tyramide signal amplification. The primary-secondary antibody complex was then stripped iteratively using heat and a low-pH citrate buffer, while the covalently bound fluorophore signal was retained in the tissue. This cycle was repeated sequentially for each target marker (CD4+, CD8a+, CD19+, EpCAM+, and IBA1+) on the same tissue section, enabling simultaneous visualization of all five immune and epithelial cell populations without cross-reactivity between staining rounds. We have clarified the staining protocol in Section 2.2.

**2. The study used lung sections from DO mice infected with M. tuberculosis. Which M. tuberculosis strain was used for infection? What inoculum size was used in this study?**

DO mice were infected with the *M. tb* Erdman strain with a retained lung dose of 25–100 colony forming units (CFU), as described in our previously published work [5]. The range of euthanasia day and lung bacterial burden for each disease group are now provided in Table 1.

**3. Did the authors quantify the bacterial burden in the lungs of the different disease groups, such as progressors, asymptomatic mice, and controllers? Was there any correlation between the spatial distribution of immune cells and the bacterial load at different stages of disease progression? Additionally, what time points were selected for analysis in this study? These details are currently missing.**

We performed a correlation analysis between spatial immune cell distribution and bacterial load, and present it in the newly added Section 3.7 and Figure 8. Spearman correlation coefficients were computed per disease group between the log mean distance of each immune cell population (CD4+ T cells, CD8a+ T cells, CD19+ B cells, IBA1+ macrophages, and EpCAM+ epithelial cells) and *M.tb* burden for each mouse. The per-group Spearman correlation coefficients (ρ) and associated p-values are provided in each panel of Figure 8. The results indicate that spatial immune organization, particularly T cell proximity, shows disease-state-specific associations with bacterial burden, with Asymptomatic mice displaying distinct spatial-bacterial relationships compared to Progressors and Controllers.

Regarding time points, tissue sections were collected at the time of euthanasia, which varied by disease outcome: Progressors were euthanized within 60 days of infection reflecting acute pulmonary TB, Controllers were euthanized after 60 days reflecting chronic pulmonary TB, with the longest surviving Controller reaching 418 days post-infection, and Asymptomatic mice showed no clinical signs of disease throughout the observation period. These details are described in Section 2.2, and Table 1.

**4. How did the authors define the asymptomatic stage of infection in the mouse model? Figure 1B shows lung scan images with granuloma segmentation during the asymptomatic stage; however, multiple granulomas appear to be present, similar to those observed in acute disease. Please clarify this point.**

We thank the reviewer for this important clarifying question. Asymptomatic mice in this study are defined by the absence of outward clinical signs of disease: they remain infected, develop granulomas, and gain weight to the same degree as non-infected controls throughout the observation period, as described in detail in our previously published work [6].

Regarding the appearance of granulomas in Figure 1B, granulomas in Asymptomatic mice may appear morphologically similar in size and shape to those in Progressors on mIF imaging. This is expected and does not reflect a classification error. The key distinguishing pathological feature of Progressor granulomas is neutrophilic infiltration and necrosis, which is not visible by immunofluorescence imaging but is clearly demonstrable by hematoxylin and eosin staining, as we have described previously [1-6]. We have clarified that in the discussion section of the manuscript.
**5. The authors used the IF-UNet++ segmentation model for image analysis. What are the advantages of this model compared with other segmentation algorithms, such as Cellpose, which has been used in similar studies (Kumar et al., 2024)?**

Cellpose and similar cell segmentation algorithms are designed to delineate individual cells, identifying and separating each cell's boundary in an image. In this study, the biological task is fundamentally different. The goal is to segment granulomas, which are entire multicellular structures composed of clusters of immune cells (T cells, B cells, macrophages, and others) organized around *M.tb*-infected tissue. These are not single cells but complex, heterogeneous tissue-level structures that can span large regions of a WSIs. Using a cell-segmentation tool like Cellpose for granuloma delineation would be a category mismatch: it would produce tens of thousands of individual cell outlines rather than a single boundary around the granuloma as a functional immunological unit.

The IF-UNet++ model was specifically designed and optimized for this granuloma-level task. It is an immunofluorescence-adapted UNet++ architecture that processes multiple immunofluorescence channels simultaneously (CD4+, CD8a+, CD19+, EpCAM+, IBA1+), leveraging all available marker information to accurately identify granuloma boundaries in complex multiplex images, whereas standard segmentation tools like Cellpose are typically optimized for brightfield or single-channel fluorescence microscopy. Furthermore, the model was trained and validated on gigapixel-scale WSIs, achieving an IoU of 93.22% on the training set and 90.57% on an independent test set, demonstrating robust generalization that would be unattainable by repurposing a cell-segmentation model. Finally, because IF-UNet++ precisely delineates the granuloma as a biological unit, all subsequent spatial analyses including T cell-macrophage proximity, B cell-epithelial cell distances, and immune cell densities are anchored to biologically meaningful boundaries, which a cell-level segmentation tool cannot provide. We have clarified that in section 2.4.1, 3.1.

**References:**

[1]	M. K. Niazi *et al.*, "Lung necrosis and neutrophils reflect common pathways of susceptibility to Mycobacterium tuberculosis in genetically diverse, immune-competent mice," *Disease models & mechanisms,* vol. 8, no. 9, pp. 1141-1153, 2015.

[2]	T. E. Tavolara *et al.*, "Automatic discovery of clinically interpretable imaging biomarkers for Mycobacterium tuberculosis supersusceptibility using deep learning," *EBioMedicine,* vol. 62, 2020.

[3]	T. E. Tavolara, M. Niazi, A. C. Gower, M. Ginese, G. Beamer, and M. N. Gurcan, "Deep learning predicts gene expression as an intermediate data modality to identify susceptibility patterns in Mycobacterium tuberculosis infected Diversity Outbred mice," *EBioMedicine,* vol. 67, 2021.

[4]	D. Koyuncu *et al.*, "CXCL1: A new diagnostic biomarker for human tuberculosis discovered using Diversity Outbred mice," *PLoS Pathogens,* vol. 17, no. 8, p. e1009773, 2021.

[5]	D. M. Gatti *et al.*, "Systems genetics uncover new loci containing functional gene candidates in Mycobacterium tuberculosis-infected Diversity Outbred mice," *PLoS Pathogens,* vol. 20, no. 6, p. e1011915, 2024.

[6]	D. Koyuncu *et al.*, "B cells in perivascular and peribronchiolar granuloma-associated lymphoid tissue and B-cell signatures identify asymptomatic Mycobacterium tuberculosis lung infection in Diversity Outbred mice," *Infection and immunity,* vol. 92, no. 7, pp. e00263-23, 2024.

[7]	M. Ahmed *et al.*, "Immune correlates of tuberculosis disease and risk translate across species," *Science translational medicine,* vol. 12, no. 528, p. eaay0233, 2020.

[8]	R. Gopal *et al.*, "S100A8/A9 proteins mediate neutrophilic inflammation and lung pathology during tuberculosis," *American journal of respiratory and critical care medicine,* vol. 188, no. 9, pp. 1137-1146, 2013.
