# Supplementary material for: Deep learning-based spatial immunoprofiling of multiplex immunofluorescence images distinguishes tuberculosis disease states in diversity outbred mice
Source: PLoS One. 2026 Sep 25;21(9):e0358334. doi: 10.1371/journal.pone.0358334 (PMC13614646; doi:10.1371/journal.pone.0358334)
Supplement: Peer review history

# Peer review: Decision Letter 0

**DOI:** 10.1371/journal.pone.0358334.r001

**Authors:** Selvakumar Subbian

15 May 2026

Dear Dr. Sajjad,

Thank you for submitting your manuscript to PLOS ONE. After careful consideration, we feel that it has merit but does not fully meet PLOS ONE’s publication criteria as it currently stands. Therefore, we invite you to submit a revised version of the manuscript that addresses the points raised during the review process.

==============================

**ACADEMIC EDITOR:**  Although the premise of this article seems innovative, a major and significant flaw in this article is the over-emphasis of the mouse lung granulomas as a prototype for human TB disease states.  This is fundamentally incorrect and leads to misconception of "disease state" defined in human TB.  It is unclear as what exactly the mouse "disease state" that correlate with the human counterpart in this study? As the authors may aware, standard mouse granulomas are strikingly different from the highly complex, heterogenous and evolved human granulomas. There is no equivalent of necrotizing, caseating, liquefied and cavitary lesions in mouse lungs infected with TB; however, these were hallmarks in human TB. For example, cavitary granulomas are associated with active TB, which is a disease state. Thus, assigning a "disease state" with mostly a cellular aggregate-type granulomas that doesn't have the crucial "maturity" of granulomas in the mice model is incorrect.  At this point, I suggest the authors to revise the title and the content of the article to align with what is factual in the mice model granulomas. For example, the authors may focus their tone towards how to improvise the spatial immunoprofiling of mouse lung granulomas, without linking any "disease state", since there is no true correlate of "disease state" in mice that represent the spectrum of outcomes in humans.  Rather the focus should be on how to gain more insight into the heterogeneous lung granulomas in the DO mice using DL-based spatial immunoprofiling. If the authors would like to challenge this view, then they should provide evidence of "disease state" in their mouse model, such as sputum positivity, x-ray scores, fever, weight loss etc., these are some of the standard diagnostic measures to evaluate the disease state in human TB.

==============================

Please submit your revised manuscript by Jun 29 2026 11:59PM. If you will need more time than this to complete your revisions, please reply to this message or contact the journal office at plosone@plos.org. When you're ready to submit your revision, log on to https://www.editorialmanager.com/pone/ and select the 'Submissions Needing Revision' folder to locate your manuscript file.

- A letter that responds to each point raised by the academic editor and reviewer(s). You should upload this letter as a separate file labeled 'Response to Reviewers'.
- A marked-up copy of your manuscript that highlights changes made to the original version. You should upload this as a separate file labeled 'Revised Manuscript with Track Changes'.
- An unmarked version of your revised paper without tracked changes. You should upload this as a separate file labeled 'Manuscript'.

If you would like to make changes to your financial disclosure, please include your updated statement in your cover letter. Guidelines for resubmitting your figure files are available below the reviewer comments at the end of this letter.

If applicable, we recommend that you deposit your laboratory protocols in protocols.io to enhance the reproducibility of your results. Protocols.io assigns your protocol its own identifier (DOI) so that it can be cited independently in the future. For instructions see: https://journals.plos.org/plosone/s/submission-guidelines#loc-laboratory-protocols. Additionally, PLOS ONE offers an option for publishing peer-reviewed Lab Protocol articles, which describe protocols hosted on protocols.io. Read more information on sharing protocols at https://plos.org/protocols?utm_medium=editorial-email&utm_source=authorletters&utm_campaign=protocols.

As the corresponding author, your ORCID iD is verified in the submission system and will appear in the published article. PLOS supports the use of ORCID, and we encourage all coauthors to register for an ORCID iD and use it as well. Please encourage your coauthors to verify their ORCID iD within the submission system before final acceptance, as unverified ORCID iDs will not appear in the published article. *Only*  the individual author can complete the verification step; PLOS staff *cannot*  verify ORCID iDs on behalf of authors.

We look forward to receiving your revised manuscript.

Kind regards,

Selvakumar Subbian, Ph.D.

Academic Editor

PLOS One

Journal Requirements:

When submitting your revision, we need you to address these additional requirements.

1. Please ensure that your manuscript meets PLOS ONE's style requirements, including those for file naming. The PLOS ONE style templates can be found at https://journals.plos.org/plosone/s/file?id=wjVg/PLOSOne_formatting_sample_main_body.pdf and https://journals.plos.org/plosone/s/file?id=ba62/PLOSOne_formatting_sample_title_authors_affiliations.pdf

2. Please note that PLOS One has specific guidelines on code sharing for submissions in which author-generated code underpins the findings in the manuscript. In these cases, we expect all author-generated code to be made available without restrictions upon publication of the work. Please review our guidelines at https://journals.plos.org/plosone/s/materials-and-software-sharing#loc-sharing-code and ensure that your code is shared in a way that follows best practice and facilitates reproducibility and reuse.

3. Thank you for stating in your Funding Statement:

[The work was supported through a National Institutes of Health (NIH) R01 HL145411 (PI: Beamer), R01 CA276301 (PIs: Niazi, Chen). The content is solely the responsibility of the authors and does not necessarily represent the official views of the National Institutes of Health.].

Please provide an amended statement that declares *all* the funding or sources of support (whether external or internal to your organization) received during this study, as detailed online in our guide for authors at http://journals.plos.org/plosone/s/submit-now.  Please also include the statement “There was no additional external funding received for this study.” in your updated Funding Statement.

Please include your amended Funding Statement within your cover letter. We will change the online submission form on your behalf.

4. Please remove any funding-related text from the manuscript. Please note that funding information should not appear in any section or other areas of your manuscript. We will publish the funding statement that is present in the Financial Disclosure section of the online submission form.

5. We note that you have indicated that there are restrictions to data sharing for this study. PLOS only allows data to be available upon request if there are legal or ethical restrictions on sharing data publicly. For more information on unacceptable data access restrictions, please see http://journals.plos.org/plosone/s/data-availability#loc-unacceptable-data-access-restrictions.

Before we proceed with your manuscript, please address the following prompts:

a) If there are ethical or legal restrictions on sharing a de-identified data set, please explain them in detail (e.g., data contain potentially identifying or sensitive patient information, data are owned by a third-party organization, etc.) and who has imposed them (e.g., a Research Ethics Committee or Institutional Review Board, etc.). Please also provide contact information for a data access committee, ethics committee, or other institutional body to which data requests may be sent.

b) If there are no restrictions, please upload the minimal anonymized data set necessary to replicate your study findings to a stable, public repository and provide us with the relevant URLs, DOIs, or accession numbers. For a list of recommended repositories, please see

https://journals.plos.org/plosone/s/recommended-repositories. You also have the option of uploading the data as Supporting Information files, but we would recommend depositing data directly to a data repository if possible.

We will update your Data Availability statement on your behalf to reflect the information you provide.

6. Please include captions for your Supporting Information files at the end of your manuscript, and update any in-text citations to match accordingly. Please see our Supporting Information guidelines for more information: http://journals.plos.org/plosone/s/supporting-information

If the reviewer comments include a recommendation to cite specific previously published works, please review and evaluate these publications to determine whether they are relevant and should be cited. There is no requirement to cite these works unless the editor has indicated otherwise.

[Note: HTML markup is below. Please do not edit.]

Reviewers' comments:

Reviewer's Responses to Questions

**Comments to the Author**

1. Is the manuscript technically sound, and do the data support the conclusions?

Reviewer #1: Yes

Reviewer #2: Yes

**********

2. Has the statistical analysis been performed appropriately and rigorously?

Reviewer #1: Yes

Reviewer #2: I Don't Know

**********

3. Have the authors made all data underlying the findings in their manuscript fully available?

The PLOS Data policy

Reviewer #1: Yes

Reviewer #2: Yes

**********

4. Is the manuscript presented in an intelligible fashion and written in standard English?

Reviewer #1: Yes

Reviewer #2: Yes

**********

Reviewer #1: In this review, the authors have devised artificial intelligence and machine learning techniques to analyze Tuberculous granulomas for better understanding of disease progression and prediction. The study highlights the significant bottlenecks in understanding Tuberculosis. However, I have following concerns regarding the manuscript,

1. The authors have not included any functional markers (e.g., M1/M2 polarization, TNFα, IL1β) for spatial localization and their significance? This is very important for coming to any conclusion.

2. Given the subjectivity in granuloma boundary definition, how do the authors ensure annotation consistency across samples?

3. Did the authors quantitatively compare predicted vs. true granuloma morphology (e.g., size, shape descriptors.

4. How does segmentation quality impact downstream biological interpretation or feature extraction.

5. The classification labels are defined by survival: Progressor (<60 days) vs. Controller (>60 days). Is this threshold biologically justified or empirically chosen? Also, how stable are these labels across cohorts? Could survival-based labeling introduce bias unrelated to granuloma morphology?

6. Please clarify how distances were computed from pixel-to-micron.

7. How were bronchiolar epithelial cells defined? Based on EpCAM positivity alone? Were airway structures (bronchioles) explicitly segmented or annotated? EpCAM+ cells may include non-bronchiolar epithelial cells unless spatially constrained.

8. Why would closer B-cell proximity to bronchiolar epithelium associated with asymptomatic infection?

9. Were analyses stratified by: Granuloma size? Necrotic vs. non-necrotic lesions? Spatial organization can vary dramatically with granuloma stage.

Reviewer #2: Authors investigated the spatial distribution of lung granulomas in different clinical stages of tuberculosis disease using a mouse model. The authors developed a novel automated model for granuloma segmentation in lung tissues. The study demonstrates distinct spatial distributions of CD4+ and CD8a+ positive cells during the asymptomatic stage of disease. In addition, the LLM model decoded complex cellular patterns within granulomas, including balanced immune responses in asymptomatic mice, dysfunctional responses with low cellularity in acute TB, and increased immune cell accumulation during chronic disease. Overall, the findings are interesting and contribute valuable insights into the immunopathology of tuberculosis.

Major Comments:

1. The authors performed multiplex immunofluorescence imaging of lung sections. Did the authors use sequential staining on the same tissue sections after stripping the previous antibodies/signals? This is not clearly described in the Methods section.

2. The study used lung sections from DO mice infected with M. tuberculosis. Which M. tuberculosis strain was used for infection? What inoculum size was used in this study?

3. Did the authors quantify the bacterial burden in the lungs of the different disease groups, such as progressors, asymptomatic mice, and controllers? Was there any correlation between the spatial distribution of immune cells and the bacterial load at different stages of disease progression? Additionally, what time points were selected for analysis in this study? These details are currently missing.

4. How did the authors define the asymptomatic stage of infection in the mouse model? Figure 1B shows lung scan images with granuloma segmentation during the asymptomatic stage; however, multiple granulomas appear to be present, similar to those observed in acute disease. Please clarify this point.

5. The authors used the IF-UNet++ segmentation model for image analysis. What are the advantages of this model compared with other segmentation algorithms, such as Cellpose, which has been used in similar studies (Kumar et al., 2024)?

**********

what does this mean?). If published, this will include your full peer review and any attached files.

If you choose “no”, your identity will remain anonymous but your review may still be made public.

**Do you want your identity to be public for this peer review?**  For information about this choice, including consent withdrawal, please see our Privacy Policy

Reviewer #1: **Yes:** Ranjeet Kumar

Reviewer #2: **Yes:** Afsal Kolloli

**********

[NOTE: If reviewer comments were submitted as an attachment file, they will be attached to this email and accessible via the submission site. Please log into your account, locate the manuscript record, and check for the action link "View Attachments". If this link does not appear, there are no attachment files.]

To ensure your figures meet our technical requirements, please review our figure guidelines: https://journals.plos.org/plosone/s/figures

You may also use PLOS’s free figure tool, NAAS, to help you prepare publication quality figures: https://journals.plos.org/plosone/s/figures#loc-tools-for-figure-preparation.

NAAS will assess whether your figures meet our technical requirements by comparing each figure against our figure specifications.

# Author comment: Author response to Decision Letter 1

**DOI:** 10.1371/journal.pone.0358334.r002

7 Jul 2026

Reviewer #1: In this review, the authors have devised artificial intelligence and machine learning techniques to analyze Tuberculous granulomas for better understanding of disease progression and prediction. The study highlights the significant bottlenecks in understanding Tuberculosis. However, I have following concerns regarding the manuscript,

1. The authors have not included any functional markers (e.g., M1/M2 polarization, TNFα, IL1β) for spatial localization and their significance? This is very important for coming to any conclusion.

We thank the reviewer for this important and valid observation. We acknowledge that the absence of functional markers such as M1/M2 polarization indicators, TNFα, and IL-1β for spatial localization is a limitation of the current study, and we have added this to the limitations section of the Discussion accordingly. The primary aim of this study was to develop and validate an automated computational framework for granuloma segmentation, disease-state classification, and spatial quantification of immune cell populations from mIF images, rather than to provide a comprehensive functional characterization of immune responses across disease states. The functional immune and inflammatory responses, bacterial control capacities, and molecular signatures of the Progressor, Asymptomatic, and Controller phenotypes have been extensively studied in our prior work and by others, using protein detection methods, gene expression profiling, and pathway analysis. Specifically, lung cytokines and chemokines including TNFα, IFN-γ, IL-10, CXCL1, CXCL2, CXCL5, MMP8, S100A8, and VEGF have been quantified and analyzed across disease states in our previously published studies [1-6]. The immune and inflammatory correlates of these disease phenotypes have also been validated by independent groups [7, 8].

The current study builds on this established functional foundation by adding a spatial dimension, quantifying where immune cells are located within granulomas and how those spatial relationships differ across disease states. The inclusion of functional markers for spatial localization in future mIF panels is an important direction that we fully agree with and acknowledge as a priority for subsequent work.

2. Given the subjectivity in granuloma boundary definition, how do the authors ensure annotation consistency across samples?

We thank the reviewer for this insightful question. The authors ensured annotation consistency by grounding the entire segmentation pipeline in expert pathological knowledge. Granuloma boundary annotations were delineated by a board-certified pathologist, providing biologically accurate and consistent ground truth segmentation masks for model training. Importantly, deep learning segmentation models learn by identifying recurring patterns in training data, meaning that inconsistent or contradictory annotations across samples would prevent the model from learning a coherent decision boundary, ultimately resulting in poor performance on unseen data. The IF-UNet++ model was then trained on these expert-defined granuloma annotations and quantitatively evaluated, achieving an IoU of 90.57% on the test set, demonstrating that the model successfully learned to replicate pathologist-level accuracy across diverse disease states and tissue morphologies. The fact that IF-UNet++ achieves high IoU on samples never seen during training is therefore not only a measure of model accuracy but also indirect quantitative evidence that the underlying annotations were consistent and reproducible across the dataset. Beyond quantitative evaluation, the segmentation results were mapped back to the original mIF images and qualitatively reviewed by a board-certified pathologist, who confirmed that the predicted granuloma boundaries were spatially concordant with true granulomatous structures across all disease phenotypes. Taken together, the expert-guided annotation, strong quantitative performance, and qualitative pathologist validation form a coherent and mutually reinforcing validation strategy that collectively addresses the inherent subjectivity in granuloma boundary definition. We have expanded this into section 3.1.

3. Did the authors quantitatively compare predicted vs. true granuloma morphology (e.g., size, shape descriptors.

We performed the quantitative comparison of predicted versus true granuloma morphology using the Intersection over Union (IoU) metric, which serves as a comprehensive surrogate for all aspects of granuloma morphological accuracy, including size, shape, spatial extent, and boundary fidelity.

IoU is computed by dividing the area of overlap between the predicted segmentation mask and the ground truth mask by the area of their union. If the predicted boundary is too large or too small, the size mismatch directly penalizes the score. If the shape is incorrect, the misaligned boundaries reduce the shared region and lower the IoU. If the spatial location is off, the same penalty applies. In this way, IoU implicitly encodes agreement in size, shape, location, and boundary fidelity all within a single unified metric, making it a comprehensive surrogate for granuloma morphological accuracy without requiring each descriptor to be reported separately.

Our IF-UNet++ model achieved an IoU of 93.22% on the training set and 90.57% on the test set, demonstrating strong spatial and morphological agreement with the pathologist-defined ground truth across diverse disease states and tissue morphologies. We therefore believe that the high IoU scores reported in our study provide sufficient evidence of accurate granuloma geometry capture, and that these results are adequate for supporting the reliability of our downstream spatial and disease-state analyses. We have further clarified it in section 3.1.

4. How does segmentation quality impact downstream biological interpretation or feature extraction.

We thank the reviewer for highlighting the importance of segmentation quality with respect to downstream biological interpretation and feature extraction. To directly address this question, we introduced controlled perturbation by deliberately shrinking the granuloma boundary by 10% of its area and recomputed downstream cellular metrics, including immune cell density per unit granuloma area and total immune cell counts across all cell types (Supplementary Figure S1, S2).

Our findings demonstrate that although the absolute value of cell counts and densities change because of the boundary perturbation, the overall biological trends across the three disease groups, namely Progressor, Asymptomatic, and Controller, remain consistent with our original results. This indicates that our downstream biological interpretations are robust to minor segmentation inaccuracies and are not artifacts of precise boundary placement. The biological signatures we report, such as higher CD4+ and CD8a+ T cell densities in Asymptomatic mice and elevated total cell counts in Controllers, are preserved under this perturbation, supporting the stability and reliability of our conclusions.

We have added the corresponding plots as Supplementary Figures S1 and S2, showing density of immune cell populations per unit granuloma area and total immune cell numbers respectively, both computed under the 10% boundary shrinkage condition, for all five cell types across the three disease states. These supplementary results collectively demonstrate that our framework produces conclusions that are resilient to small variations in segmentation quality.

A. CD4 B. CD8a

C. CD19 D. IBA1

E. EPCAM

Supplementary Figure S1. Density of immune cell populations per unit granuloma area across TB disease states (10% boundary shrinkage applied). Box plots show density of cells, reported as density per unit granuloma area for (A) CD4+ T cells, (B) CD8a+ T cells, (C) CD19+ B cells, (D) IBA1+ macrophages, and (E) EpCAM+ epithelial cells across three disease groups: Progressor, Asymptomatic, and Controller. Each dot represents one granuloma; box plots show median, interquartile range, and whiskers extending to 1.5× IQR.

A. CD4 B. CD8a

C. CD19 D. IBA1

E. EPCAM

Supplementary Figure S2. Total numbers of immune cells in granulomas across TB disease states (10% boundary shrinkage applied). Box plots showing cell abundance for (A) CD4+ T cells, (B) CD8a+ T cells, (C) CD19+ B cells, (D) IBA1+ macrophages, and (E) EpCAM+ epithelial cells across three disease groups: Progressor, Asymptomatic TB, and Controller. Each dot (4A-4E) represents one granuloma; box plots show median, interquartile range, and whiskers extending to 1.5× IQR.

5. The classification labels are defined by survival: Progressor (<60 days) vs. Controller (>60 days). Is this threshold biologically justified or empirically chosen? Also, how stable are these labels across cohorts? Could survival-based labeling introduce bias unrelated to granuloma morphology?

The 60-day survival threshold is biologically justified and empirically well-supported across our cohorts. As described in our previously published work [5], across nearly 1,000 total DO mice spanning cohorts of approximately 125 mice each, 25–30% consistently develop pulmonary TB morbidity requiring euthanasia prior to day 60, with a peak morbidity wave occurring between days 25–35 post-infection. The remaining 70–75% are asymptomatic through this 60-day window and many remain so substantially beyond that, up to nearly 2 years. This survival pattern across independent cohorts demonstrates that the 60-day threshold captures a real and stable biological boundary between acute disease susceptibility and bacterial control, rather than an arbitrary cutoff.

We acknowledge that survival-based labeling could theoretically introduce bias if mice developed morbidity due to non-TB disease. This was minimized by examining all mice through multiple approaches to confirm that morbidity was related to pulmonary TB and not to other co-morbidities. Mice with clear co-morbidities were excluded and are not included in any analyses presented here. The detailed methods used to ensure that morbidity was attributable to TB rather than other disease are described in the Quantification of TB-related Traits (Phenotyping) section of our previously published work [5].

6. Please clarify how distances were computed from pixel-to-micron.

We appreciate the reviewer raising this point. Our WSIs have a spatial resolution of 0.5 microns per pixel (mpp), meaning each pixel corresponds to a physical length of 0.5 µm in tissue space. To ensure biologically meaningful spatial analysis, all pixel-based distance measurements were converted to physical units using this linear scaling factor. Specifically, to identify cell populations within a 75 µm radius, we converted the radius from microns to pixels using the formula: RadiusInPixels = RadiusInMicrons / mpp = 75 / 0.5 = 150 pixels. This conversion was applied uniformly across all WSIs to maintain consistency in defining spatial neighborhoods. The resulting 150-pixel radius was then used to query and count cell populations within that spatial boundary for each cell of interest. This approach ensures that our proximity-based analysis reflects true physical distances in tissue, independent of any pixel-resolution variability across imaging sessions. We have clarified it in the manuscript section 3.5.

7. How were bronchiolar epithelial cells defined? Based on EpCAM positivity alone? Were airway structures (bronchioles) explicitly segmented or annotated? EpCAM+ cells may include non-bronchiolar epithelial cells unless spatially constrained.

EpCAM positive cells were confirmed to be bronchiolar epithelial cells based on the anatomic structure highlighted by board certified pathologists. We have clarified it in section 3.2.

8. Why would closer B-cell proximity to bronchiolar epithelium associated with asymptomatic infection?

This is a good question that we did not address directly here but could be an association or a mechanism. A plausible hypothesis is that B-cells in that region are differentiating into plasma cells that may secrete IgA into the airways or IgG into the blood stream that may help the host control infection in the lungs or limit dissemination.

9. Were analyses stratified by: Granuloma size? Necrotic vs. non-necrotic lesions? Spatial organization can vary dramatically with granuloma stage.

Analyses were stratified by host disease state. We agree that stratification by other means is a good idea and spatial organization can vary by granuloma stage. These types of questions may be better answered by separate experiments using inbred strains of mice at pre-defined time points.

Reviewer #2: Authors investigated the spatial distribution of lung granulomas in different clinical stages of tuberculosis disease using a mouse model. The authors developed a novel automated model for granuloma segmentation in lung tissues. The study demonstrates distinct spatial distributions of CD4+ and CD8a+ positive cells during the asymptomatic stage of disease. In addition, the LLM model decoded complex cellular patterns within granulomas, including balanced immune responses in asymptomatic mice, dysfunctional responses with low cellularity in acute TB, and increased immune cell accumulation during chronic disease. Overall, the findings are interesting and contribute valuable insights into the immunopathology of tuberculosis.

Major Comments:

1. The authors performed multiplex immunofluorescence imaging of lung sections. Did the authors use sequential staining on the same tissue sections after stripping the previous antibodies/signals? This is not clearly described in the Methods section.

We use tyramide signaling amplification for our multiplex immunofluorescence images. In each staining cycle, a primary-secondary antibody complex was applied, followed by covalent deposition of the fluorophore onto the tissue via tyramide signal amplification. The primary-secondary antibody complex was then stripped iteratively using heat and a low-pH citrate buffer, while the covalently bound fluorophore signal was retained in the tissue. This cycle was repeated sequentially for each target marker (CD4+, CD8a+, CD19+, EpCAM+, and IBA1+) on the same tissue section, enabling simultaneous visualization of all five immune and epithelial cell populations without cross-reactivity between staining rounds. We have clarified the staining protocol in Section 2.2.

2. The study used lung sections from DO mice infected with M. tuberculosis. Which M. tuberculosis strain was used for infection? What inoculum size was used in this study?

DO mice were infected with the M. tb Erdman strain with a retained lung dose of 25–100 colony forming units (CFU), as described in our previously published work [5]. The range of euthanasia day and lung bacterial burden for each disease group are now provided in Table 1.

3. Did the authors quantify the bacterial burden in the lungs of the different disease groups, such as progressors, asymptomatic mice, and controllers? Was there any correlation between the spatial distribution of immune cells and the bacterial load at different stages of disease progression? Additionally, what time points were selected for analysis in this study? These details are currently missing.

We performed a correlation analysis between spatial immune cell distribution and bacterial load, and present it in the newly added Section 3.7 and Figure 8. Spearman correlation coefficients were computed per disease group between the log mean distance of each immune cell population (CD4+ T cells, CD8a+ T cells, CD19+ B cells, IBA1+ macrophages, and EpCAM+ epithelial cells) and M.tb burden for each mouse. The per-group Spearman correlation coefficients (ρ) and associated p-values are provided in each panel of Figure 8. The results indicate that spatial immune organization, particularly T cell proximity, shows disease-state-specific associations with bacterial burden, with Asymptomatic mice displaying distinct spatial-bacterial relationships compared to Progressors and Controllers.

Regarding time points, tissue sections were collected at the time of euthanasia, which varied by disease outcome: Progressors were euthanized within 60 days of infection reflecting acute pulmonary TB, Controllers were euthanized after 60 days reflecting chronic pulmonary TB, with the longest surviving Controller reaching 418 days post-infection, and Asymptomatic mice showed no clinical signs of disease throughout the observation period. These details are described in Section 2.2, and Table 1.

4. How did the authors define the asymptomatic stage of infection in the mouse model? Figure 1B shows lung scan images with granuloma segmentation during the asymptomatic stage; however, multiple granulomas appear to be present, similar to those observed in acute disease. Please clarify this point.

We thank the reviewer for this important clarifying question. Asymptomatic mice in this study are defined by the absence of outward clinical signs of disease: they remain infected, develop granulomas, and gain weight to the same degree as non-infected controls throughout the observation period, as described in detail in our previously published work [6].

Regarding the appearance of granulomas in Figure 1B, granulomas in Asymptomatic mice may appear morphologically similar in size and shape to those in Progressors on mIF imaging. This is expected and does not reflect a classification error. The key distinguishing pathological feature of Progressor granulomas is neutrophilic infiltration and necrosis, which is not visible by immunofluorescence imaging but is clearly demonstrable by hematoxylin and eosin staining, as we have described previously [1-6]. We have clarified that in the discussion section of the manuscript.

5. The authors used the IF-UNet++ segmentation model for image analysis. What are the advantages of this model compared with other segmentation algorithms, such as Cellpose, which has been used in similar studies (Kumar et al., 2024)?

Cellpose and similar cell segmentation algorithms are designed to delineate individual cells, identifying and separating each cell's boundary in an image. In this study, the biological task is fundamentally different. The goal is to segment granulomas, which are entire multicellular structures composed of clusters of immune cells (T cells, B cells, macrophages, and others) organized around M.tb-infected tissue. These are not single cells but complex, heterogeneous tissue-level structures that can span large regions of a WSIs. Using a cell-segmentation tool like Cellpose for granuloma delineation would be a category mismatch: it would produce tens of thousands of individual cell outlines rather than a single boundary around the granuloma as a functional immunological unit.

The IF-UNet++ model was specifically designed and optimized for this granuloma-level task. It is an immunofluorescence-adapted UNet++ architecture that processes multiple immunofluorescence channels simultaneously (CD4+, CD8a+, CD19+, EpCAM+, IBA1+), leveraging all available marker information to accurately identify granuloma boundaries in complex multiplex images, whereas standard segmentation tools like Cellpose are typically optimized for brightfield or single-channel fluorescence microscopy. Furthermore, the model was trained and validated on gigapixel-scale WSIs, achieving an IoU of 93.22% on the training set and 90.57% on an independent test set, demonstrating robust generalization that would be unattainable by repurposing a cell-segmentation model. Finally, because IF-UNet++ precisely delineates the granuloma as a biological unit, all subsequent spatial analyses including T cell-macrophage proximity, B cell-epithelial cell distances, and immune cell densities are anchored to biologically meaningful boundaries, which a cell-level segmentation tool cannot provide. We have clarified that in section 2.4.1, 3.1.

References:

[1] M. K. Niazi et al., "Lung necrosis and neutrophils reflect common pathways of susceptibility to Mycobacterium tuberculosis in genetically diverse, immune-competent mice," Disease models & mechanisms, vol. 8, no. 9, pp. 1141-1153, 2015.

[2] T. E. Tavolara et al., "Automatic discovery of clinically interpretable imaging biomarkers for Mycobacterium tuberculosis supersusceptibility using deep learning," EBioMedicine, vol. 62, 2020.

[3] T. E. Tavolara, M. Niazi, A. C. Gower, M. Ginese, G. Beamer, and M. N. Gurcan, "Deep learning predicts gene expression as an intermediate data modality to identify susceptibility patterns in Mycobacterium tuberculosis infected Diversity Outbred mice," EBioMedicine, vol. 67, 2021.

[4] D. Koyuncu et al., "CXCL1: A new diagnostic biomarker for human tuberculosis discovered using Diversity Outbred mice," PLoS Pathogens, vol. 17, no. 8, p. e1009773, 2021.

[5] D. M. Gatti et al., "Systems genetics uncover new loci containing functional gene candidates in Mycobacterium tuberculosis-infected Diversity Outbred mice," PLoS Pathogens, vol. 20, no. 6, p. e1011915, 2024.

[6] D. Koyuncu et al., "B cells in perivascular and peribronchiolar granuloma-associated lymphoid tissue and B-cell signatures identify asymptomatic Mycobacterium tuberculosis lung infection in Diversity Outbred mice," Infection and immunity, vol. 92, no. 7, pp. e00263-23, 2024.

[7] M. Ahmed et al., "Immune correlates of tuberculosis disease and risk translate across species," Science translational medicine, vol. 12, no. 528, p. eaay0233, 2020.

[8] R. Gopal et al., "S100A8/A9 proteins mediate neutrophilic inflammation and lung pathology during tuberculosis," American journal of respiratory and critical care medicine, vol. 188, no. 9, pp. 1137-1146, 2013.

Attachment Submitted filename: resp_to_editor_and_reviewers.docx

# Peer review: Decision Letter 1

**DOI:** 10.1371/journal.pone.0358334.r003

**Authors:** Selvakumar Subbian

10 Aug 2026

Dear Dr. Sajjad,

Thank you for submitting your manuscript to PLOS ONE. After careful consideration, we feel that it has merit but does not fully meet PLOS ONE’s publication criteria as it currently stands. Therefore, we invite you to submit a revised version of the manuscript that addresses the points raised during the review process.

==============================

**ACADEMIC EDITOR:**

1). Change/Expand the title for section 2.4. This is confusing with the title of Main Section-2.

2). In Section 2.2. Clarify if the 48mIF lug tissue sections are from 48 animals?.  How are these animals selected within their respective cohort. i.e., Did the controllers at 61 days have same tissue pathology and/or cellular distribution as that at 418 days?.  Explain why these extremes were included in data analysis, despite their inherent heterogeneity? .  There’s a discrepency in information between text in Section 2.2 and Table-1. Controllers were euthanized at an average of 255 days (not 60 days as put in the text). Same with progressors and asymptomatic animals. How long are the asymptomatic animals monitored? Make the statements very precise and consistent.  In the “limitations of the study” section of discussion, add the skewed sample selection from prior studies as a weakness. Also, did you use “blinded mIF sections” to independently validate the performance of your DL/LLM-based system? If not, add that as a limitation.

3). In Figure-8, add the unit for lung Mtb burden in the X-axis.

4). The IACUC (animal) and IBC (Mtb) protocols were approved by Tuft University; however, no author or acknowledgment has been provided. This is an important Ethical component; therefore, clarify this and provide clear details.

5). Just be cautious about the claim that “this is the first study to combine mIF, attention-based MIL, and LLM-driven interpretation to distinguish TB disease states at the level of the granuloma in a genetically diverse animal model” in the introduction. I suggest modifying this statement.

Take this opportunity to fully-revise the manuscript for clarity, readability and tyops.

==============================

Please submit your revised manuscript by Sep 24 2026 11:59PM. If you will need more time than this to complete your revisions, please reply to this message or contact the journal office at plosone@plos.org. When you're ready to submit your revision, log on to https://www.editorialmanager.com/pone/ and select the 'Submissions Needing Revision' folder to locate your manuscript file.

- A letter that responds to each point raised by the academic editor and reviewer(s). You should upload this letter as a separate file labeled 'Response to Reviewers'.
- A marked-up copy of your manuscript that highlights changes made to the original version. You should upload this as a separate file labeled 'Revised Manuscript with Track Changes'.
- An unmarked version of your revised paper without tracked changes. You should upload this as a separate file labeled 'Manuscript'.

If you would like to make changes to your financial disclosure, please include your updated statement in your cover letter. Guidelines for resubmitting your figure files are available below the reviewer comments at the end of this letter.

If applicable, we recommend that you deposit your laboratory protocols in protocols.io to enhance the reproducibility of your results. Protocols.io assigns your protocol its own identifier (DOI) so that it can be cited independently in the future. For instructions see: https://journals.plos.org/plosone/s/submission-guidelines#loc-laboratory-protocols. Additionally, PLOS ONE offers an option for publishing peer-reviewed Lab Protocol articles, which describe protocols hosted on protocols.io. Read more information on sharing protocols at https://plos.org/protocols?utm_medium=editorial-email&utm_source=authorletters&utm_campaign=protocols.

As the corresponding author, your ORCID iD is verified in the submission system and will appear in the published article. PLOS supports the use of ORCID, and we encourage all coauthors to register for an ORCID iD and use it as well. Please encourage your coauthors to verify their ORCID iD within the submission system before final acceptance, as unverified ORCID iDs will not appear in the published article. *Only*  the individual author can complete the verification step; PLOS staff *cannot*  verify ORCID iDs on behalf of authors.

We look forward to receiving your revised manuscript.

Kind regards,

Selvakumar Subbian, Ph.D.

Academic Editor

PLOS One

Journal Requirements:

1. If the reviewer comments include a recommendation to cite specific previously published works, please review and evaluate these publications to determine whether they are relevant and should be cited. There is no requirement to cite these works unless the editor has indicated otherwise.

2. Please review your reference list to ensure that it is complete and correct. If you have cited papers that have been retracted, please include the rationale for doing so in the manuscript text, or remove these references and replace them with relevant current references. Any changes to the reference list should be mentioned in the rebuttal letter that accompanies your revised manuscript. If you need to cite a retracted article, indicate the article’s retracted status in the References list and also include a citation and full reference for the retraction notice.

[Note: HTML markup is below. Please do not edit.]

Reviewers' comments:

Reviewer's Responses to Questions

**Comments to the Author**

Reviewer #1: All comments have been addressed

Reviewer #2: All comments have been addressed

**********

2. Is the manuscript technically sound, and do the data support the conclusions?

Reviewer #1: Yes

Reviewer #2: Yes

**********

3. Has the statistical analysis been performed appropriately and rigorously?

Reviewer #1: Yes

Reviewer #2: I Don't Know

**********

4. Have the authors made all data underlying the findings in their manuscript fully available?

The PLOS Data policy

Reviewer #1: Yes

Reviewer #2: Yes

**********

5. Is the manuscript presented in an intelligible fashion and written in standard English?

Reviewer #1: Yes

Reviewer #2: Yes

**********

Reviewer #1: (No Response)

Reviewer #2: (No Response)

**********

what does this mean?). If published, this will include your full peer review and any attached files.

If you choose “no”, your identity will remain anonymous but your review may still be made public.

**Do you want your identity to be public for this peer review?**  For information about this choice, including consent withdrawal, please see our Privacy Policy

Reviewer #1: **Yes:** Ranjeet Kumar

Reviewer #2: No

**********

[NOTE: If reviewer comments were submitted as an attachment file, they will be attached to this email and accessible via the submission site. Please log into your account, locate the manuscript record, and check for the action link "View Attachments". If this link does not appear, there are no attachment files.]

To ensure your figures meet our technical requirements, please review our figure guidelines: https://journals.plos.org/plosone/s/figures

You may also use PLOS’s free figure tool, NAAS, to help you prepare publication quality figures: https://journals.plos.org/plosone/s/figures#loc-tools-for-figure-preparation.

NAAS will assess whether your figures meet our technical requirements by comparing each figure against our figure specifications.

# Author comment: Author response to Decision Letter 2

**DOI:** 10.1371/journal.pone.0358334.r004

26 Aug 2026

Dear Editor,

We sincerely thank you for your careful evaluation of our manuscript and for the constructive comments provided during this revision. In response, we have revised the manuscript to clarify the composition, selection, and timing of the Diversity Outbred mouse cohort; explicitly acknowledge cohort heterogeneity, retrospective sample selection, and the lack of an independent blinded validation cohort; clarify the ethical approvals and provenance of the archived specimens; add the appropriate units to Figure 8; and moderate the novelty-related language. We have also carefully reviewed the manuscript throughout to improve clarity, consistency, readability, and grammatical accuracy.

Detailed responses to the Reviewers are below.

Sincerely,

Usama Sajjad, on behalf of the authors

==============================

ACADEMIC EDITOR: The authors must address the following concerns directly on the manuscript,

1). Change/Expand the title for section 2.4. This is confusing with the title of Main Section-2.

We thank the reviewer for this helpful suggestion. In response to the comment, we have revised the title of Section 2.4 to more clearly distinguish it from the main Materials and Methods section and to better reflect the content of the subsection. The revised title is: “Computational Framework for Granuloma Segmentation, Disease-State Classification, and Spatial Interpretation.”

2). In Section 2.2. Clarify if the 48mIF lug tissue sections are from 48 animals?. How are these animals selected within their respective cohort. i.e., Did the controllers at 61 days have same tissue pathology and/or cellular distribution as that at 418 days?. Explain why these extremes were included in data analysis, despite their inherent heterogeneity? . There’s a discrepency in information between text in Section 2.2 and Table-1. Controllers were euthanized at an average of 255 days (not 60 days as put in the text). Same with progressors and asymptomatic animals. How long are the asymptomatic animals monitored? Make the statements very precise and consistent. In the “limitations of the study” section of discussion, add the skewed sample selection from prior studies as a weakness. Also, did you use “blinded mIF sections” to independently validate the performance of your DL/LLM-based system? If not, add that as a limitation.

Thank you for this detailed and important comment. We have revised Section 2.2 to more precisely describe the composition, disease-state definitions, and timing of sample collection in the study cohort. First, we clarify that the 48 mIF lung tissue sections analyzed in this study were obtained from 48 individual DO mice, with one lung tissue section representing each animal (15 Progressors, 17 Asymptomatic mice, and 16 Controllers). Samples for mIF analysis were selected to capture the full range of bacterial burden and survival outcomes within each disease class while maintaining relatively balanced groups for downstream analysis.

We also clarify that the previously stated 60-day time point represents the established phenotypic threshold used to distinguish Progressors from Controllers, rather than the average euthanasia time of either group. Specifically, in the mIF cohort analyzed here, Progressors were euthanized between 22 and 51 days post-infection (mean = 27.05 days), whereas Controllers were euthanized between 114 and 418 days post-infection (mean = 255 days). Asymptomatic mice were euthanized at predetermined time points between 48 and 50 days post-infection (mean = 49 days) and remained clinically asymptomatic during this observation period. These definitions are consistent with the previously established disease-state criteria in which Progressors develop TB-related morbidity before 60 days, Controllers develop chronic pulmonary TB after surviving beyond 60 days, and Asymptomatic animals remain clinically healthy until their predetermined euthanasia time point. We have revised the text to make these distinctions explicit and consistent with Table 1.

Regarding the broad survival duration among Controllers, these animals were grouped according to their previously established chronic pulmonary TB phenotype rather than according to a fixed post-infection collection time. We agree, however, that the wide range in disease duration may introduce biological heterogeneity within the Controller group. The present study was not designed to determine whether cellular composition or spatial organization remains unchanged across different durations of chronic disease; therefore, we have avoided making such an assumption and now explicitly acknowledge this temporal heterogeneity as a limitation.

The specimens used for mIF represented a retrospectively selected subset of archived samples from the previously characterized cohorts. Because the study used an archived subset rather than a prospectively selected cohort, we have also acknowledged the possibility of selection bias in the Discussion.

Finally, an entirely independent, blinded set of mIF sections was not available for validation of the present framework. Model performance was evaluated using five-fold cross-validation with held-out test samples within the available cohort. We agree that evaluation using an independently collected and blinded mIF cohort would provide a stronger assessment of generalizability, and we have added this point to the limitations of the study.

3). In Figure-8, add the unit for lung Mtb burden in the X-axis.

We thank the reviewer for this helpful suggestion. In response to the reviewer’s comment, we carefully reviewed all figures to ensure that the appropriate units are clearly indicated wherever applicable. In particular, for Figure 8, the unit for lung M. tb burden has now been added to improve clarity and consistency.

A B

C D

E

Figure 8. Correlation between spatial distribution of immune cells and M.tb burden across disease states. (A). Log mean distance for CD4+ T cells (B). CD8a+ T cells, (C). CD19+ B cells, (D). IBA1+ macrophages, and (E). EPCAM+ epithelial cells plotted against M.tb burden for each mouse. Each point represents one mouse, colored by disease group: Asymptomatic (blue), Controllers (green), and Progressors (red). Per-group Spearman correlation coefficients (ρ) and associated p-values are shown in each panel legend.

4). The IACUC (animal) and IBC (Mtb) protocols were approved by Tuft University; however, no author or acknowledgment has been provided. This is an important Ethical component; therefore, clarify this and provide clear details.

Thank you for highlighting this important ethical consideration. We apologize that the relationship between the archived samples and the Tufts University-approved animal studies was not sufficiently clear. The lung tissue samples analyzed in the present study were generated during previously conducted M. tb infection experiments at Tufts University. These experiments were performed under Tufts University IACUC protocols G2012-53, G2015-33, G2018-33, and G2020-121 and IBC registrations GRIA04, GRIA10, GRIA17, and 2020-G61. We have revised the Ethics Statement to explicitly clarify the provenance of these archived specimens and their relationship to the approved protocols. We also note that the Tufts University biocontainment personnel and facilities involved in these studies are acknowledged in the manuscript.

5). Just be cautious about the claim that “this is the first study to combine mIF, attention-based MIL, and LLM-driven interpretation to distinguish TB disease states at the level of the granuloma in a genetically diverse animal model” in the introduction. I suggest modifying this statement.

We thank the reviewer for the great suggestion. In response to the review comments, we have softened the tone of our language and modified the highlighted claim to “Overall, this study integrates mIF imaging, attention-based MIL, and LLM-driven interpretation to characterize granuloma-level cellular and spatial patterns associated with distinct TB disease states in a genetically diverse animal model.”

Take this opportunity to fully-revise the manuscript for clarity, readability and tyops.

==============================

Please submit your revised manuscript by Sep 24 2026 11:59PM. If you will need more time than this to complete your revisions, please reply to this message or contact the journal office at plosone@plos.org. When you're ready to submit your revision, log on to https://www.editorialmanager.com/pone/ and select the 'Submissions Needing Revision' folder to locate your manuscript file.

Please include the following items when submitting your revised manuscript:

• A letter that responds to each point raised by the academic editor and reviewer(s). You should upload this letter as a separate file labeled 'Response to Reviewers'.

• A marked-up copy of your manuscript that highlights changes made to the original version. You should upload this as a separate file labeled 'Revised Manuscript with Track Changes'.

• An unmarked version of your revised paper without tracked changes. You should upload this as a separate file labeled 'Manuscript'.

If you would like to make changes to your financial disclosure, please include your updated statement in your cover letter. Guidelines for resubmitting your figure files are available below the reviewer comments at the end of this letter.

If applicable, we recommend that you deposit your laboratory protocols in protocols.io to enhance the reproducibility of your results. Protocols.io assigns your protocol its own identifier (DOI) so that it can be cited independently in the future. For instructions see: https://journals.plos.org/plosone/s/submission-guidelines#loc-laboratory-protocols. Additionally, PLOS ONE offers an option for publishing peer-reviewed Lab Protocol articles, which describe protocols hosted on protocols.io. Read more information on sharing protocols at https://plos.org/protocols?utm_medium=editorial-email&utm_source=authorletters&utm_campaign=protocols.

As the corresponding author, your ORCID iD is verified in the submission system and will appear in the published article. PLOS supports the use of ORCID, and we encourage all coauthors to register for an ORCID iD and use it as well. Please encourage your coauthors to verify their ORCID iD within the submission system before final acceptance, as unverified ORCID iDs will not appear in the published article. Only the individual author can complete the verification step; PLOS staff cannot verify ORCID iDs on behalf of authors.

We look forward to receiving your revised manuscript.

Kind regards,

Selvakumar Subbian, Ph.D.

Academic Editor

PLOS One

Journal Requirements:

1. If the reviewer comments include a recommendation to cite specific previously published works, please review and evaluate these publications to determine whether they are relevant and should be cited. There is no requirement to cite these works unless the editor has indicated otherwise.

2. Please review your reference list to ensure that it is complete and correct. If you have cited papers that have been retracted, please include the rationale for doing so in the manuscript text, or remove these references and replace them with relevant current references. Any changes to the reference list should be mentioned in the rebuttal letter that accompanies your revised manuscript. If you need to cite a retracted article, indicate the article’s retracted status in the References list and also include a citation and full reference for the retraction notice.

[Note: HTML markup is below. Please do not edit.]

Reviewers' comments:

Reviewer's Responses to Questions

Comments to the Author

1. If the authors have adequately addressed your comments raised in a previous round of review and you feel that this manuscript is now acceptable for publication, you may indicate that here to bypass the “Comments to the Author” section, enter your conflict of interest statement in the “Confidential to Editor” section, and submit your "Accept" recommendation.

Reviewer #1: All comments have been addressed

Reviewer #2: All comments have been addressed

________________________________________

2. Is the manuscript technically sound, and do the data support the conclusions?

The manuscript must describe a technically sound piece of scientific research with data that supports the conclusions. Experiments must have been conducted rigorously, with appropriate controls, replication, and sample sizes. The conclusions must be drawn appropriately based on the data presented.

Reviewer #1: Yes

Reviewer #2: Yes

________________________________________

3. Has the statistical analysis been performed appropriately and rigorously?

Reviewer #1: Yes

Reviewer #2: I Don't Know

________________________________________

4. Have the authors made all data underlying the findings in their manuscript fully available?

The PLOS Data policy requires authors to make all data underlying the findings described in their manuscript fully available without restriction, with rare exception (please refer to the Data Availability Statement in the manuscript PDF file). The data should be provided as part of the manuscript or its supporting information, or deposited to a public repository. For example, in addition to summary statistics, the data points behind means, medians and variance measures should be available. If there are restrictions on publicly sharing data—e.g. participant privacy or use of data from a third party—those must be specified.

Reviewer #1: Yes

Reviewer #2: Yes

________________________________________

5. Is the manuscript presented in an intelligible fashion and written in standard English?

PLOS ONE does not copyedit accepted manuscripts, so the language in submitted articles must be clear, correct, and unambiguous. Any typographical or grammatical errors should be corrected at revision, so please note any specific errors here.

Reviewer #1: Yes

Reviewer #2: Yes

________________________________________

6. Review Comments to the Author

Please use the space provided to explain your answers to the questions above. You may also include additional comments for the author, including concerns about dual publication, research ethics, or publication ethics. (Please upload your review as an attachment if it exceeds 20,000 characters)

Reviewer #1: (No Response)

Reviewer #2: (No Response)

________________________________________

7. PLOS authors have the option to publish the peer review history of their article (what does this mean?). If published, this will include your full peer review and any attached files.

If you choose “no”, your identity will remain anonymous but your review may still be made public.

Do you want your identity to be public for this peer review? For information about this choice, including consent withdrawal, please see our Privacy Policy.

Reviewer #1: Yes: Ranjeet Kumar

Reviewer #2: No

________________________________________

[NOTE: If reviewer comments were submitted as an attachment file, they will be attached to this email and accessible via the submission site. Please log into your account, locate the manuscript record, and check for the action link "View Attachments". If this link does not appear, there are no attachment files.]

To ensure your figures meet our technical requirements, please review our figure guidelines: https://journals.plos.org/plosone/s/figures

You may also use PLOS’s free figure tool, NAAS, to help you prepare publication quality figures: https://journals.plos.org/plosone/s/figures#loc-tools-for-figure-preparation.

NAAS will assess whether your figures meet our technical requirements by comparing each figure against our figure specifications.

________________________________________

In compliance with data protection regulations, you may request that we remove your personal registration details at any time. (Remove my information/details). Please contact the publication office if you have any questions.

# Editor report: Decision Letter 2

**DOI:** 10.1371/journal.pone.0358334.r005

**Authors:** Selvakumar Subbian

31 Aug 2026

Deep Learning-Based Spatial Immunoprofiling of Multiplex Immunofluorescence Images Distinguishes Tuberculosis Disease States in Diversity Outbred Mice

PONE-D-26-15268R2

Dear Dr. Sajjad,

We’re pleased to inform you that your manuscript has been judged scientifically suitable for publication and will be formally accepted for publication once it meets all outstanding technical requirements.

Within one week, you’ll receive an e-mail detailing the required amendments. When these have been addressed, you’ll receive a formal acceptance letter and your manuscript will be scheduled for publication.

An invoice will be generated when your article is formally accepted. Please note, if your institution has a publishing partnership with PLOS and your article meets the relevant criteria, all or part of your publication costs will be covered. Please make sure your user information is up-to-date by logging into Editorial Manager at Editorial Manager® and clicking the ‘Update My Information' link at the top of the page. For questions related to billing, please contact billing support.

If your institution or institutions have a press office, please notify them about your upcoming paper to help maximize its impact. If they’ll be preparing press materials, please inform our press team as soon as possible -- no later than 48 hours after receiving the formal acceptance. Your manuscript will remain under strict press embargo until 2 pm Eastern Time on the date of publication. For more information, please contact onepress@plos.org.

Kind regards,

Selvakumar Subbian, Ph.D.

Academic Editor

PLOS One

Additional Editor Comments (optional):

Reviewers' comments:

# Editor report: Acceptance letter

**DOI:** 10.1371/journal.pone.0358334.r006

**Authors:** Selvakumar Subbian

PONE-D-26-15268R2

PLOS One

Dear Dr. Sajjad,

I'm pleased to inform you that your manuscript has been deemed suitable for publication in PLOS One. Congratulations! Your manuscript is now being handed over to our production team.

At this stage, our production department will prepare your paper for publication. This includes ensuring the following:

* All references, tables, and figures are properly cited

* All relevant supporting information is included in the manuscript submission,

* There are no issues that prevent the paper from being properly typeset

You will receive further instructions from the production team, including instructions on how to review your proof when it is ready. Please keep in mind that we are working through a large volume of accepted articles, so please give us a few days to review your paper and let you know the next and final steps.

Lastly, if your institution or institutions have a press office, please let them know about your upcoming paper now to help maximize its impact. If they'll be preparing press materials, please inform our press team within the next 48 hours. Your manuscript will remain under strict press embargo until 2 pm Eastern Time on the date of publication. For more information, please contact onepress@plos.org.

You will receive an invoice from PLOS for your publication fee after your manuscript has reached the completed accept phase. If you receive an email requesting payment before acceptance or for any other service, this may be a phishing scheme. Learn how to identify phishing emails and protect your accounts at https://explore.plos.org/phishing.

If we can help with anything else, please email us at customercare@plos.org.

Thank you for submitting your work to PLOS One and supporting open access.

Kind regards,

PLOS One Editorial Office Staff

on behalf of

Dr. Selvakumar Subbian

Academic Editor

PLOS One
